# Atlas2 Cloud: a framework for personal genome analysis in the cloud

**DOI:** 10.1186/1471-2164-13-S6-S19

**Published:** 2012-10-26

**Authors:** Uday S Evani, Danny Challis, Jin Yu, Andrew R Jackson, Sameer Paithankar, Matthew N Bainbridge, Adinarayana Jakkamsetti, Peter Pham, Cristian Coarfa, Aleksandar Milosavljevic, Fuli Yu

**Affiliations:** 1 The Human Genome Sequencing Center, Baylor College of Medicine, Houston, TX 77030, USA; 2 Bioinformatics Research Laboratory, Epigenome Center, Department of Molecular and Human Genetics, Baylor College of Medicine, TX 77030, USA; 3 Department of Molecular and Human Genetics, Baylor College of Medicine, TX 77030, USA

## Abstract

**Background:**

Until recently, sequencing has primarily been carried out in large genome centers which have invested heavily in developing the computational infrastructure that enables genomic sequence analysis. The recent advancements in next generation sequencing (NGS) have led to a wide dissemination of sequencing technologies and data, to highly diverse research groups. It is expected that clinical sequencing will become part of diagnostic routines shortly. However, limited accessibility to computational infrastructure and high quality bioinformatic tools, and the demand for personnel skilled in data analysis and interpretation remains a serious bottleneck. To this end, the cloud computing and Software-as-a-Service (SaaS) technologies can help address these issues.

**Results:**

We successfully enabled the Atlas2 Cloud pipeline for personal genome analysis on two different cloud service platforms: a community cloud via the Genboree Workbench, and a commercial cloud via the Amazon Web Services using Software-as-a-Service model. We report a case study of personal genome analysis using our Atlas2 Genboree pipeline. We also outline a detailed cost structure for running Atlas2 Amazon on whole exome capture data, providing cost projections in terms of storage, compute and I/O when running Atlas2 Amazon on a large data set.

**Conclusions:**

We find that providing a web interface and an optimized pipeline clearly facilitates usage of cloud computing for personal genome analysis, but for it to be routinely used for large scale projects there needs to be a paradigm shift in the way we develop tools, in standard operating procedures, and in funding mechanisms.

## Background

The revolutionary development of massively parallel DNA sequencing has enabled identification of biomedically relevant genomic variants via whole genome [1] and exome resequencing [2]. Information relevant for personalized medicine such as assessment of longitudinal disease risks, and personalized treatment [3] are now within reach.

In a few very recent personal genomic studies, results have directly led to targeted treatment and dramatic improvement in the patient's quality of life [4]. These examples are paving the way to soon turn genomic sequencing into a routine diagnostic procedure and enable personalized medicine.

Currently, analysis of sequencing data on a genomic scale requires bioinformatic expertise and access to extensive computational resources, presenting a significant barrier. Most cutting-edge genome analysis applications [5,6] are still limited to a command line interface and require at least moderate informatics expertise to operate. In addition, large scale genomic data analysis requires routine access to a high performance compute cluster. Such requirements are entirely unsuitable for the operational models of smaller research/diagnostic laboratories due to the excessive investment requirements in computing infrastructure and personnel.

The deployment of genomic analysis Software as a Service (SaaS) within a cloud computing framework offers a unique solution for these problems. The concept behind cloud computing is to outsource computation to third-party servers or clusters at a remote location. This allows small laboratories to take advantage of external computational resources without having to maintain an in-house compute cluster. This software as a service model removes the upfront investment requirement and any delays associated with building local computing infrastructure. Earlier solutions such as CloudBurst [7] and Crossbow [8] have attempted to tackle the very specific problem of mapping short read data and assembling large genomes using the scalability offered by the map-reduce framework deployed on top of a compute cluster. While this is useful the users would still need to have considerable bioinformatics skill and acquaintance with cluster infrastructure to undertake such an analysis. Other solutions such as CloudMan [9] from the Galaxy Project provide a user interface and remove the need for user to have informatics experience but are not specifically designed for personal genome analysis.

To this end we integrated our variant analysis pipeline - Atlas2 Suite - onto a "local cloud" using the Genboree Workbench http://www.genboree.org and onto a "commercial cloud" via the Amazon Web Services http://aws.amazon.com. We performed a case study using the Atlas2 Genboree pipeline as a proof of concept to demonstrate the potential of personal genome analysis on the cloud. We also processed two whole exome capture samples using our Atlas2 Amazon pipeline to outline the cost of running analysis on Amazon. Our cloud analysis pipeline on Genboree has a web browser-based drag and drop interface, allowing users to interact with the software through their browser at any location, and making it practical for the software to be used by non-bioinformaticians. Our cloud pipeline is actively maintained by our team, which also removes the need for users to update the software.

## Methods

### Deploying the Atlas2 personal genomic analysis pipeline via the Genboree Workbench (Atlas2 Genboree)

The Atlas2 Suite is a variant detection software package optimized for variant discovery in exome capture data on all the three next generation sequencing platforms [10] (Roche 454, Illumina and SOLiD). The suite consists of Atlas-SNP2 for calling Single Nucleotide Polymorphisms (SNPs) and Atlas-Indel2 for calling short INsertions and DELetions (INDELs) http://www.hgsc.bcm.tmc.edu/cascade-tech-software-ti.hgsc. These tools have been available for command line usage, and applied to a number of large scale projects including the International 1000 Genomes Project [11], The Cancer Genome Atlas Project (TCGA), and follow-up resequencing in the context of disease genome wide association studies.

**Figure 1:**
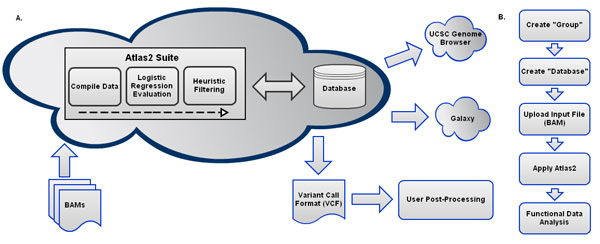
A: High level representation of the Atlas2 Genboree pipeline. Figure 1B: Specific steps involved in running Atlas2 Genboree.

Genboree Workbench is a platform for deploying genomic tools as a service and is deployed at Baylor College of Medicine http://www.genboree.org. The Genboree Workbench Graphical User Interfaces (GUI) extensively relies on Ext-JS, a JavaScript library. Tools within the workbench make API (Application Programming Interface) calls to the REST (REpresentational State Transfer) API which is hosted on a thin server. This is done asynchronously using Asynchronous JavaScript and XML (AJAX). Since Genboree System uses REST style of architecture to communicate between the server and the client, it allowed us to easily integrate Atlas2 within a couple of weeks. Genboree is backed by a small cluster of nodes which are managed by the TORQUE resource manager (an open source tool) and Maui (developed by Adaptive Computing) to schedule jobs. Atlas2 Genboree can be accessed as a Genboree Workbench Toolset. Users from external groups with access to a web browser can 1) upload data onto the cloud, 2) run Atlas2 for variant analysis, and 3) visualize the variant calling results using different genome browsers such as the Genboree Browser or University of California, Santa Cruz Genome Browser[12] (http://www.genome.ucsc.edu) (Figure 1A). Atlas2 Genboree has a web-interface with hierarchical click-through steps. The self-explanatory nature of the web-interface eases the usage overhead. The workflow illustrated in Figure 1B shows the specific steps in running the Atlas2 Suite on the Genboree System.

#### Genboree Data Selector

**Figure 2:**
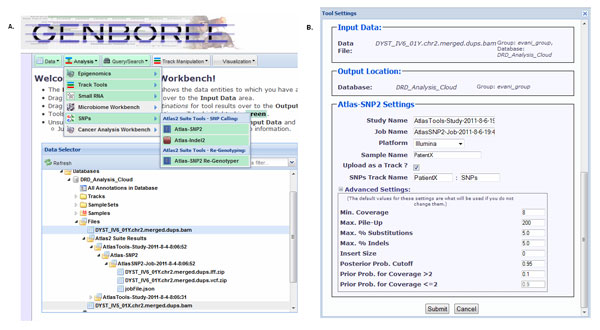
A: Shows how various folders are represented within Genboree, and how to navigate the menu bar to get to Atlas2 Suite. Figure 2B: Atlas2 Suite customization window.

The Genboree Workbench organizes data in a hierarchal tree. Before using the Atlas2 Suite users must define a group and create a database. Within the database are the "Files" and "Tracks" subdirectories. Files contain input files uploaded by the user and output files generated by Atlas2. Tracks contain processed output files which can be used for visualization on the Genboree browser. This hierarchical representation is shown in a screenshot of the Genboree workbench in Figure 2A.

#### Uploading data onto Atlas2 Genboree

Atlas2 Genboree accepts Binary sequence Alignment/Mapping format (BAM) files as input. Files are uploaded onto Genboree by dragging the destination database from the Data Selector to the Output Targets box and selecting "transfer files" under the data tab in the menu. A prompt window allows users to select an input BAM file from their local computer and upload it to the cloud servers. A 24 GB BAM file took approximately one hour to upload on a 50 Mb/sec bandwidth connection.

#### Variant calling

The Atlas2 Suite may be run by simply assigning the desired input and output and selecting the appropriate tool (Figure 2A). Atlas2 Genboree allows users to specify parameter cutoffs in the job parameter-setting window (Figure 2B). Here one can choose from the three different sequencing platforms and tune the parameters.

The tool produces two output files, an LFF file and a Variant Call Format (VCF) [13] file which are stored under the files section inside of the database specified in the output target box. The LFF format is adapted from the LDAS upload format used to store variants and annotations http://www.genboree.org/java-bin/showHelp.jsp?topic=lffFileFormat. Both the files can be downloaded by selecting the specific file and clicking on the download file option.

### Genboree system allows integration with third party tools

Cloud deployment may produce "silos" of integration where extension of analysis pipelines and addition of analysis steps beyond those offered as a service may be hard to accomplish. To overcome this problem, Genboree system provides application programming interfaces for programmatic access to all the data and tools. Also data is accessible in formats that can be readily fed into a variety of ancillary tools. The interfaces and data format compatibilities enable mixing-and-matching of tools required in specific steps such as visualization in various genome browsers including UCSC genome browser, invocation of pipelines such as Galaxy [14], and integration with custom or third-party variant analysis and annotation tools such as ANNOVAR[15]. As described next, we successfully tested all three types of integration.

#### Visualizing variants with genome browsers

##### Genboree browser

The variant calls can be readily viewed in the Genboree genome browser. After going into the browser, variants can be visualized by selecting the appropriate database. Genboree browser supports looking at variants from multiple samples simultaneously.

##### UCSC genome browser

The variants called by Atlas2 Genboree can be directly exported to UCSC genome browser [16] for further viewing, annotation and analysis. The variants can be exported by converting our variants file into a BigBed format file (http://genome.ucsc.edu/goldenPath/help/bigBed.html) via the cloud file conversion functionality.

#### Integration with Galaxy

As our initial trial, we were able to upload our raw VCF file downloaded from Genboree without post-processing onto Galaxy and convert the VCF file into a multiple alignment format (MAF) custom track using the VCF to MAF custom track function with Graph/Display data.

#### Post-processing with third party variant annotation tools

The VCF file downloaded from Genboree was annotated and filtered using ANNOVAR. ANNOVAR categorizes variants into intronic, exonic, splicing, non-coding RNA, 5' untranslated region, 3' untranslated region, upstream, downstream and intergenic. The exonic variants are further categorized into synonymous, nonsynonymous, stop gain (gain of stop function), stop lost (loss of stop function), and frameshift or non-frameshift changes caused by insertions, deletions or block substitutions. ANNOVAR can also be used to filter out variants found in dbSNP.

### Enabling the Atlas2 personal genomic analysis pipeline via Amazon Web Services (Atlas2 Amazon)

The Amazon Web Services (AWS) provides virtualized computational infrastructure on demand. AWS can be tailored to provide scalable and flexible solutions for application hosting, web applications and high performance computing. We used the Amazon Elastic Compute Cloud (EC2) and Amazon Simple Storage Service (S3) to enable Atlas2 on Amazon. The EC2 allows users to lease a wide variety of EC2 instances which differ based on the amount of compute nodes and memory (http://aws.amazon.com/ec2/#instance). Amazon S3 is a persistent data storage solution offered by AWS, which is meant to be highly scalable and have low latency. Both the EC2 and S3 services can be managed from the AWS Management Console.

**Figure 3:**
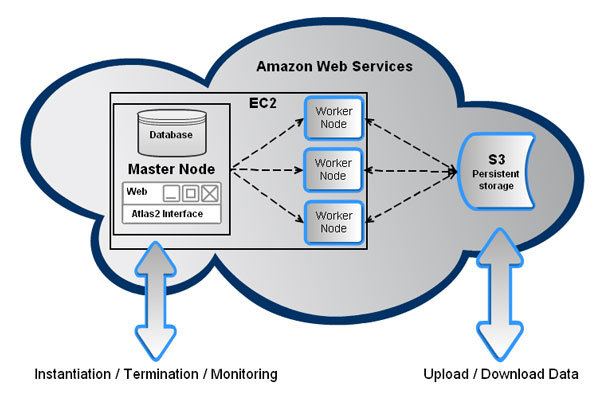
Schematic representation of the Atlas2 Amazon pipeline.

Our Atlas2 cloud pipeline on AWS was designed ground up to be specifically used for personal genome analysis. The web user interface, developed using the Spring Framework written in Java, provides access to Atlas2 suite on the machine image; this user friendly interface can be used to submit jobs and monitor worker nodes (Figure 3). The application runs on Apache Tomcat (version 5.5.35) and can be accessed through port 8080. The backend code, on the Atlas2 machine image, is optimized to efficiently analyze data and ease the process of adding newer tools to the pipeline in the context of genome analysis. The backend code was written in Python (version 2.7.2). Fabric (version 1.4.1), Amazon EC2 API Tools (version 1.4.3 2011-05-15) and s3cmd (version 1.0.0) are integral part of the backend code. Fabric was used for executing commands on the worker nodes, Amazon EC2 API Tools were used to start, terminate and monitor the status of worker instances and s3cmd was used to interact with the S3. Figure 3 provides an overview of the Atlas2 Amazon pipeline.

In order to access the Atlas2 cloud pipeline the user has to first register for an AWS account by going to http://aws.amazon.com/. Once registered the user needs to sign up for EC2 and S3 services. The user then starts an instance using the public Atlas2 machine image (ami-2ee23847) which can be found inside the community Amazon Machine Image (AMI) tab. Before starting the instance the user needs to change his Security Groups setting so as to enable http access on port 8080 to be able to access the webpage which can be done using the public DNS of the instance which can be found in AWS management console. Since the master instance is only acting as a portal to access and monitor the jobs running on AWS this master instance can be a "t1.micro" instance. The advantage of a "t1.micro" instance is they are cheap and at the time of writing this article every new registered AWS user would get 750 hours of free runtime every month for a year; afterwards it is $0.02/hour.

**Figure 4:**
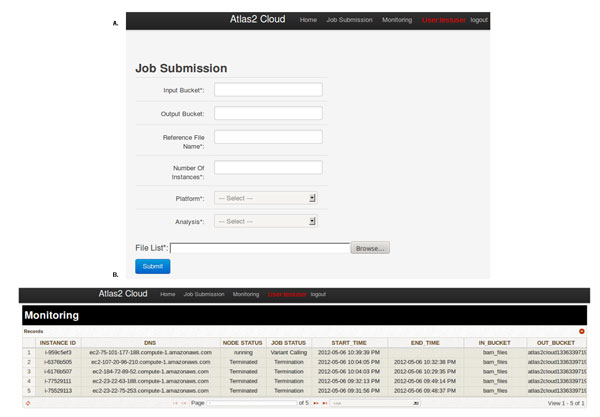
A: Screenshot of the submissions page. Figure 4B: Screenshot of the monitoring page.

Once able to access the webpage the user must create an account before they can access the pipeline. By way of creating an account, this instance can support multiple users and users do not have to type in AWS credentials each time they submit a job. The AWS credentials are needed to start additional instances and to access user data on S3. To submit a job users must provide the name of the folder on S3 containing the input files and reference FASTA needed for analysis, maximum number of parallel EC2 instances to run, upload a file with the list of input files to be processed, reference file name, sequencing platform and analysis to be performed. Figure 4A shows a screenshot of the job submissions page. Currently Atlas2 Amazon expects the user to upload the data onto S3; this can be done by going to the S3 tab on AWS management console. Alternatively, users may take advantage of the AWS Import/Export option wherein the user can ship a portable storage device to Amazon and it will securely process and transfer the data onto S3. This option is extremely useful in uploading large amounts of data to S3 due to network bandwidth bottleneck.

The monitoring page shows information regarding each worker node, i.e. each row in the monitoring page represents a worker instance. For each worker instance the following information is shown; instance id, public DNS, node status, job status, start time, end time, input bucket and output bucket. The instance id and the public DNS can be used to access the worker node. Node status can have two possible states either the worker node is running or has been terminated. Job status can have five possible states, started refers to worker node has been instantiated, setup refers to head node is prepping the worker instance, downloading reference suggests that the worker instance is downloading the reference FASTA file, variant calling suggests worker node is running the analysis and termination refers to there are no more jobs in the queue and worker node is being terminated. Figure 4B shows a screenshot of the monitoring page.

## Results

### Applying the Altas2 Genboree to a case of personal genome study

We tested Atlas2 Genboree by performing an analysis on a recently published personal whole genome sequencing data set [4]. We examined the resource usage metrics and reproducibility in variant analysis, and examined the challenges related to integrating multiple tools required for variant detection, visualization, and analysis.

#### Description of the personal genome data set

Bainbridge et al.[4] employed the SOLiD 4 next-generation sequencing platform, and sequenced the complete genomes of a 14-year-old fraternal twin pair, one female (patientX) and one male (patientY) diagnosed with dopa (3,4-dihydrophenylalanine)-responsive dystonia (DRD). DRD is a genetically heterogeneous and clinically complex movement disorder with parkinsonian features that is usually treated with L-dopa. After identifying six heterozygous autosomal mutations in three genes, a new clinical intervention was prescribed that dramatically improved the quality of life of both twins.

#### Variant analysis

We analyzed Chromosomes 2 and 19 since all six mutations were on these two chromosomes. Uploading the BAM files, ~ 22 GB in size, took ~70 minutes using the Genboree workbench interface. We ran Atlas2 using SNP default settings for the SOLiD platform. It took an average of ~6 hours to run Chromosome 2 and ~ 30 minutes to run Chromosome 19 (Table 1). The average memory usage on the cloud node was ~800 MB. Detailed numbers of time taken to upload, run Atlas-SNP2 and memory usage are summarized in Table 1. The VCF file generated on the cloud were then downloaded for further analysis.

**Table 1:** Summarizes the amount of computation and time required to get the data (Chr 2 and Chr 19) onto Genboree and to run through the variant calling steps.

	Resource usage (Chr2/Chr 19)	
	
	PatientX	PatientY
Size of BAM file (GB)	22/5	24/6
Time to upload (Min)	70/12	85/13
Atlas2 Runtime (Min)	390/27	420/33
Atlas2 Memory Usage (MB)	1196/275	1192/270

Combining results from the Chromosome 2 and 19, Atlas2 called 229,484 and 235,450 high confidence single nucleotide variants (SNV) in patientX and patientY, respectively. Annotating the VCF file using ANNOVAR we found 87.9% and 88.6% of SNV called in patientX and patientY respectively overlapped with dbSNP (v129), which is very similar to what had been found by Bainbridge et al. (88.1%, 88.7%) [4]. The annotations generated using ANNOVAR, were used to filter variants such that we could get to novel nonsynonymous SNVs which are more likely to be causal (Table 2).

**Table 2:** Summarizes the total number of raw variants found in chromosome 2 and 19 of the two patients.

	Variant calls	
	
Nucleotide Variants	PatientX	PatientY
All Variants	229484	235450
%dbSNP	87.51	87.66
Coding	1867	2062
Nonsynonymous	921	983
Coding (novel)	170	198
Nonsynonymous (novel)	124	129
Candidate genes	5	6

Raw variants were then filtered with dbSNP (ver 129) and annotated with genetic information.

Our Atlas2 pipeline successfully called all the six variants relevant in three genes in patientY whereas only five of six variants were called in patientX. Information regarding the three genes, variants and whether it was called is summarized in Table 3. The one undetected mutation by our pipeline was in *SPR *gene at position 72969094 (A>G) causing a change from Arginine to Glycine. The reason Atlas-SNP2 was not able to call this SNV was due to a default heuristic filter which requires at least two high quality reads with variants. After examining the raw BAM file, we found that only one such variant read was found at this locus. In cases such as this, in order to lower the detection threshold, users can go back to the settings window and lower our heuristic cutoffs to achieve much higher sensitivity.

**Table 3:** Following three genes were found to contain two or more predicted amino acid altering heterozygous mutation in both the patients.

				Reproducibility	
				
Chr	Position	Reference/Variant	Gene	PatientX	PatientY
2	72972139	A/T	*SPR*	Found	Found
2	72969094	A/G	*SPR*	Not Found	Found
19	63464322	A/G	*ZNF544*	Found	Found
19	63464133	C/A	*ZNF544*	Found	Found
2	27657528	C/T	*C2orf16*	Found	Found
2	27655701	G/C	*C2orf16*	Found	Found

The table shows how many of those mutations we reproduced.

### Applying Atlas2 Amazon to whole exome capture data

**Figure 5:**
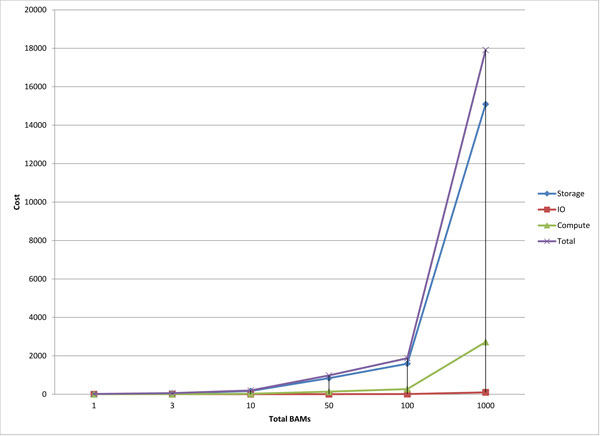
Graph based on Table 5 projecting the cost of storage, I/O and compute as we scale up data.

We ran Atlas-INDEL2 on two BAM files, one Illumina and one SOLiD, using Atlas2 Amazon, and outlined the cost of running Atlas2 Amazon to generate INDEL calls on whole exome capture data. The SOLiD and Illumina BAM files were obtained from the 1000 Genomes phase 1 project they were 35GB and 14GB in size respectively and took 3 and 1 hours to upload to S3 using the AWS management console. The Illumina BAM (NA19093, YRI) contained ~135 Million reads with average read lengths of 75bp and the SOLiD BAM (HG00099, GBR) contained ~247 Million reads with average read lengths of 50bp the average depth coverage across the capture region in both the BAM files was ~30X. The processing was done on an "m1.large" EC2 instance which comes with 2 Elastic Compute Units (ECU), 7.5 GB of memory and 850GB of local instance storage. It took us ~11 hours and ~8 hours at a cost of $8.67 and $4.85 to process SOLiD and Illumina BAM files respectively. A detailed breakdown of the cost incurred on storage, compute and I/O on the attached EBS volume is summarized in Table 4. Based on our experiences with Amazon we projected the cost of running Atlas2 Amazon on 3, 10, 50, 100 and 1000 BAM files the data is shown in Table 5. While trying to make the projections we tried to be as realistic as possible, but had to make a few assumptions. We assumed average size of BAM files to be 20GB and storage cost was computed for a 6 month period. Compute cost was based on "m1.large" EC2 instance which has a running cost of $0.34/hour and 1 million I/O requests per BAM. Graph depicting the cost projection can be seen in Figure 5.

**Table 4:** Summarizes the cost of running Atlas-INDEL2 on whole exome capture SOLiD and Illumina BAMs using Atlas2 Amazon.

	SOLiD	Illumina
**Time to upload (Hours)**	3	1

**Size of BAM (GB)**	34.6	13.9
**I/O (Millions)**	0.9	1.8
**Compute (Hours)**	10.5	7.7

**Storage Cost**	4.84	1.95
**I/O Cost**	0.09	0.18
**Compute Cost**	3.74	2.72

**Total Cost (USD)**	**8.67**	**4.85**

**Table 5:** Following table summarizes the cost projections of analyzing 1, 3, 10, 50, 100 and 1000 BAMs using Atlas2 Amazon.

No of BAM	1	3	10	50	100	1000
**Size (GB)**	20	60	200	1000	2000	20000
**I/O (Millions)**	1	3	10	50	100	1000
**Compute time (Hrs)**	8	24	80	400	800	8000

**Storage Cost**	16.8	50.4	168	840	1592.16	15092.16
**I/O Cost**	0.1	0.3	1	5	10	100
**Compute Cost**	2.72	8.16	27.2	136	272	2720

**Total Cost (USD)**	19.62	58.86	196.2	981	1874.16	17912.16

## Conclusions

If personal genomic studies are to become a routine part of personalized diagnostics and medical management that is accessible to small research and clinical laboratories, advanced bioinformatic analysis must be made accessible both in terms of computational resources and usability. We have demonstrated the suitability of deploying existing analysis tools onto a cloud resource to address these issues, and demonstrated its utility by duplicating a real-world case study of clinical significance. We also outlined the cost of running Atlas2 Amazon pipeline on SOLiD and Illumina whole exome capture samples and made cost projections of running the analysis on much larger sample sizes.

These analyses show that Atlas2 on Genboree and Amazon provide both possible and practical solution for personal genome variant analysis on the cloud by outsourcing the computation resources and expertise needed to perform such analysis. By removing these barriers, Atlas2 on the cloud enables non-bioinformaticians at small research labs to perform this analysis without the need to invest in expensive compute clusters. It is our hope that various pipelines will have output that is cross-compatible with each other so as to enable and facilitate the creation of customized personal genomic analysis.

While the present Atlas2 Amazon architecture and AWS cost structure are certainly a viable solution for small scale personal genome analysis the storage cost in the long run can make it prohibitive for large scale analysis. With the growing number of competitors in the cloud computing space we believe the cost of storage and compute is eventually going to come down and by harnessing the power of distributed computing algorithms like map-reduce framework will make it attractive for large scale analysis. There are other serious consideration such as data security which is of utmost importance especially in a clinical setting, the burden of which lies in the hands of both developers and end users and until such issues are resolved they pose a serious hindrance for clinical use. Other minor issues include the network-bandwidth bottleneck, but this is a onetime problem since once the data is uploaded onto the cloud it can used for multiple analyses. Once these challenges have been addressed we believe that genome analysis on the cloud will become a valuable resource, enabling both large and small scale clinical analysis by a variety of diverse research groups.

## Abbreviations

AWS: Amazon Web Services; EC2: Amazon Elastic Compute Cloud; S3: Amazon Simple Storage Service; EBS: Amazon Elastic Block Storage; ECU: Elastic Compute Unit; AMI: Amazon Machine Image; SNV: Single Nucleotide Variants; INDEL: Insertions and Deletions; BAM: Binary sequence Alignment/Map format; VCF: Variant Call Format; YRI: Yoruba in Ibadan Nigeria; GBR: British from England and Scotland.

## Availability and requirements

The Atlas2-Cloud machine image is made public and can be instantiated from the AWS management console by searching for the following Amazon machine image ID ami-ec469c85. Since machine image IDs are not permanent and susceptible to change when we update the machine image the better way to find the Atlas2 image would be to search for "atlas2" in the community AMI tab. The Atlas2-Amazon backend source code is released under the BSD license and is available for download at http://sourceforge.net/projects/atlas2cloud/ . More detailed instructions and tutorial on how to access the pipeline can be found at our Sourceforge page.

## Competing interests

AM is a founder and owns shares in IP Genesis, Inc., a company that owns commercial licensing rights to Genboree. USE, DC, JY, ARJ, SP, MNB, AJ, PP, CC and FY declare they have no conflicts of interest.

## Authors' contributions

FY and AM conceived and directed the project. DC, JY and USE developed Atlas2 Suite. ARJ, SP, CC and AM developed and maintain Genboree Workbench, and integrated the Atlas2 Suite with it. USE, AJ and PP enabled Atlas2 on Amazon. MNB provided us with clinical data. USE and FY designed and carried out the analysis. USE, DC, AM and FY prepared the manuscript.
